# The effect of levothyroxine treatment on left ventricular function in subclinical hypothyroidism

**DOI:** 10.20945/2359-3997000000052

**Published:** 2018-08-01

**Authors:** Valentina Velkoska Nakova, Brankica Krstevska, Elizabeta Srbinovska Kostovska, Olivija Vaskova, Ljubica Georgievska Ismail

**Affiliations:** 1 University “Goce Delchev” Faculty of Medical Science Clinical Hospital Shtip R. Macedonia University “Goce Delchev”, Faculty of Medical Science, Clinical Hospital, Shtip, R. Macedonia; 2 University Clinic of Endocrinology, Diabetes and Metabolic Disorders Medical Faculty R. Macedonia University Clinic of Endocrinology, Diabetes and Metabolic Disorders, Medical Faculty, Skopje, R. Macedonia; 3 University Clinic of Cardiology Medical Faculty R. Macedonia University Clinic of Cardiology, Medical Faculty, Skopje, R. Macedonia; 4 Institute of Patophysiology and Nuclear Medicine Medical Faculty R. Macedonia Institute of Patophysiology and Nuclear Medicine, Medical Faculty, Skopje, R. Macedonia; 5 University Clinic of Cardiology Medical Faculty R. Macedonia University Clinic of Cardiology, Medical Faculty, Skopje, R. Macedonia

**Keywords:** Subclinical hypothyroidism, thyroid replacement therapy, systolic function, diastolic function

## Abstract

**Objective:**

Treatment of subclinical hypothyroidism (ScH), especially the mild form of ScH, is controversial because thyroid hormones influence cardiac function. We investigate left ventricular systolic and diastolic function in ScH and evaluate the effect of 5-month levothyroxine treatment.

**Subjects and methods:**

Fifty-four patients with newly diagnosed mild ScH (4.2 <TSH < 10.0 mU/L) and 30 euthyroid subjects matched by age were analysed. Laboratory analyses and an echocardiography study were done at the first visit and after 5 months in euthyroid stage in patients with ScH.

**Results:**

Compared to healthy controls, patients with ScH had a lower E/A ratio (1.03 ± 0.29 vs. 1.26 ± 0.36, p < 0.01), higher E/e' sep. ratio (762 ± 2.29 vs. 6.04 ± 1.64, p < 0.01), higher myocardial performance index (MPI) (0.47 ± 0.08 vs. 0.43 ± 0.07, p < 0.05), lower global longitudinal strain (GLS) (-19.5 ± 2.3 vs. −20.9 ± 1.7%, p < 0.05), and lower S wave derived by tissue Doppler imaging (0.077 ± 0.013 vs. 0.092 ± 0.011 m/s, p < 0.01). Levothyroxine treatment in patients with ScH contributed to higher EF (62.9 ± 3.9 vs. 61.6 ± 4.4%, p < 0.05), lower E/e' sep. ratio (6.60 ± 2.06 vs. 762 ± 2.29, p < 0.01), lower MPI (0.43 ± 0.07 vs. 0.47 ± 0.08%, p < 0.01), and improved GLS (-20.07 ± 2.7 vs. −19.55 ± 2.3%, p < 0.05) compared to values in ScH patients at baseline. Furthermore, in all study populations (ScH patients before and after levothyroxine therapy and controls), TSH levels significantly negatively correlated with EF (r = −0.15, p < 0.05), E/A (r = −0.14, p < 0.05), GLS (r = −0.26, p < 0.001), and S/TDI (r = −0.22, p < 0.01) and positively correlated with E/e' sep. (r = 0.14, p < 0.05).

**Conclusion:**

Patients with subclinical hypothyroidism versus healthy individuals had subtle changes in certain parameters that indicate involvement of systolic and diastolic function of the left ventricle. Although the values of the parameters were in normal range, they were significantly different compared to ScH and the control group at baseline, as well as to the ScH groups before and after treatment.The results of our study suggest that patients with ScH must be followed up during treatment to assess improvement of the disease. Some of the echocardiography obtained parameters were reversible after levothyroxine therapy.

## INTRODUCTION

Subclinical hypothyroidism (ScH) is defined as a condition with a slight increase in serum concentration of thyrotropin (TSH) with normal values of circulating thyroid hormones, free thyroxine (FT4) and triiodothyronine (FT3).

The upper limit of TSH from which thyroid replacement therapy should start is still a topic of debate (1,2). Treatment is generally recommended in severe ScH (TSH value above 10 mU/L) (2-6). When the TSH value is less than 10 mU/L, treatment may be indicated in relation to age, presence of goiter, or antithyroid antibodies. One meta-analysis (7) proposed starting with thyroid replacement therapy when TSH levels are above 7 mU/L. Previous research (8) showed increased risk of atherosclerosis in patients with ScH at levels above 7 mU/L.

Thyroid hormones influence cardiac function (9,10). They regulate the transcription of structural and regulatory proteins in the cardiovascular system (11), predispose chronic inflammation and tissue changes (collagen alteration, dehydration), and also cause hemodynamic changes through their effect on smooth muscles in the arterial wall (10,12). All these can alter cardiac function and together with atherosclerosis increase the risk of cardiovascular manifestations.

Research has revealed that substitution therapy with levothyroxine in ScH with TSH values above 10 mU/L could improve left ventricular (LV) function (13-16). Whether the same is true for mild ScH (TSH values less than 10 mU/L) is unknown.

The aim of the study was to evaluate the effect of levothyroxine therapy on LV systolic and diastolic function in patients with a mild form of ScH (4.2 < TSH < 10.0 mU/L) and after 5 months in euthyroid stage, using two-dimensional echocardiography, pulse Doppler, tissue Doppler, and two-dimensional speckle tracking imaging. An additional aim of the study was to predict which parameters derived by echocardiography can be used to monitor patients with ScH.

## SUBJECTS AND METHODS

### Patients

The prospective study was conducted at the University Clinic of Endocrinology and the University Clinic of Cardiology in Skopje, R. Macedonia. The study included a control group and patients with a newly diagnosed mild form of ScH. The control group consisted of 30 healthy euthyroid patients (normal FT4, FT3, and TSH of 0.2-4.2 mU/L). The criteria for a diagnosis of mild ScH were 4.2 mU/L < TSH < 10.0 mU/L with normal serum FT4 10.3-24.45 pmol/L and FT3 4.2-8.1 pmol/L. According to the recommendations of the British Thyroid Association, patients with ScH were placed on thyroid replacement therapy if one of the following criteria were present: at least three signs or symptoms of hypothyroidism, positive anti-TPO antibodies and positive anti-Tg antibodies, positive family history of thyroid disease, and thyroid enlargement or goiter on ultrasonography (17). The starting dose of L-thyroxine was 25 μg. TSH was measured every 8 weeks for dose adjustment. After 5 months in continuous euthyroid state, echocardiography was repeated. The euthyroid state was achieved with a mean dose of 60.8 ± 19 μg in mean duration of 7.5 ± 2.2 months. During a period of three years, 54 consecutive patients were included in the ScH group.

The control group was included to compare echocardiography parameters from the mild form of SCH at baseline.

### Exclusion criteria

Patients with a previous history of thyroid disease receiving therapy for thyroid or cardiovascular function were not included. Patients who were cigarette smokers or who had cardiovascular disease, hypertension, hypothalamic-pituitary disease, depression, psychosis, bipolar disorders, diabetes, chronic pancreatitis, hepatic or renal disease, ovulatory dysfunction, infertility, or pregnancy were also not included.

### Ethical aspects

All patients gave informed consent to participate in the study after careful explanation of the testing protocol. The study was conducted in accordance with the Declaration of Helsinki and approved by the Ethics Committee at the Medical Faculty in Skopje, R. Macedonia.

## METHODS

At the first visit to the University Clinic of Endocrinology, blood samples for TSH, FT4, and FT3 were taken from all patients. At the same time, at the Outpatient Department of Cardiology Clinic, transthoracic echocardiography was done. Two-dimensional (2D) echocardiography, pulsed wave (PW) Doppler, tissue Doppler imaging (TDI), and 2D speckle tracking echocardiography were used to assess left ventricular systolic and diastolic function.

### Laboratory tests

All blood samples for thyroid hormones were collected from the antecubital vein between 08.00 and 09.00 a.m. TSH, FT4, and FT3 were determined by the super-sensitive chemiluminescent immunoassay (Immulite 2000, Siemens Medical Solutions Diagnostics, Los Angeles, CA, USA). The functional sensitivity for TSH was 0.004 μIU/mL, for FT4 0.3 ng/dL, and FT3 0.4 ng/dL.

### Echocardiographic measurements

Standard assessments of LV dimensions and wall thickness were performed in standard views on commercially available equipment (Vivid 7, GE) according to the joint recommendations of the American Society of Echocardiography and the European Association of Cardiovascular Imaging (18). LV volume and ejection fraction were calculated using the biplane method of disks (modified Simpson's rule). Left atrial volume was derived by the biapical area-length method and indexed to body surface area (LAVI) (18). Diastolic parameters were obtained by pulsed Doppler by placing a sample volume at the point of touching the mitral leaflets: early mitral flow (E wave, in m/s) and late mitral flow (A wave, m/s), their ratio (E/A, m/s), and deceleration time (DT, msec). Pulmonary venous flow was analysed using PW Doppler. We estimate peak systolic flow (S wave, m/s), early diastolic flow (d wave, m/s), s/d ratio, and atrial reversal flow (Ar, m/s), including Ar-A duration (msec) difference (19). PW TDI was performed in the apical 4-chamber view to assess annular early and late diastolic velocities (19). The recording was performed at a sweep speed of 100 mm/s at end-expiratory apnea. The septal, lateral, and average early diastolic velocities were measured, then the ratio of mitral flow E wave and TDI e’ wave (E/e’) for each of these annular velocities was calculated. By TDI we also estimate S wave like a maximal systolic velocity (m/sec) which is a parameter for evaluation of longitudinal systolic function (20). With the values of isovolumetric contractile time (IVCT, msec), isovolumetric relaxation time (IVRT, msec), and ejection time (ET, msec) derived by TDI, we calculated the myocardial performance index (MPI), which is a parameter for global left systolic function. We use the formula MPI=IVCT+IVRT/ET . The average of three consecutive cardiac cycles was taken for measurement of each echocardiographic index.

Global and regional peak systolic longitudinal strain was assessed from apical 2-chamber, 4-chamber, and long-axis views using speckle tracking analysis (21). Recordings were processed using an acoustic tracking software (EchoPac, GE, USA), allowing offline semiautomated analysis of speckle-based strain. All images were recorded with a high frame rate (> 50 frames/s). A semiautomatic myocardial tracking system was used, with manual correction of the endocardial border in end-systole and manual adjustment of the region of interest, if needed. The end of systole was defined as the point of aortic valve closure. The software automatically detected the frame-to-frame movement of the natural ultrasound reflecting markers (speckles) on standard ultrasonic images in two dimensions. The LV was divided into 17 segments, and each segment was analysed individually. Only myocardial segments considered to be of adequate quality by both the automatic system and the operator were included in the analysis. Global longitudinal strain for the LV was automatically provided as the average value of the regional peak systolic longitudinal strain of the three apical views by the software.

### Statistical analysis

Categorical parameters were summarized as percentages and continuous parameters as mean ± SD. The normal distribution of variables was verified with the Shapiro-Wilk test. As the distribution was normal, Student's independent *t*-test was used for the comparison of the quantitative data between the control group and the ScH group at baseline. Student's paired t-test was used for the comparison of the quantitative data before and after the levothyroxine therapy in the ScH group. For the comparison of categorical variables the chi-square test, Yates correction was used. The correlation between the tested parameters was determined using the Pearson correlation. All data analysis was performed using SPSS version 14.0 (IBM SPSS, Inc., Chicago, Illinois) and p value ≤ 0.05 was considered significant.

## RESULTS

Patients and normal controls were well matched for age, sex, BMI, and BSA. Heart rate was similar in both groups, but significantly increased in the ScH group after the levothyroxine treatment. The systolic blood pressure was significantly higher in the ScH group at baseline than in the control group, but the treatment with levothyroxine didn't make any changes. The diastolic blood pressure was similar in both groups, without difference after levothyroxine treatment. As expected, TSH levels were significantly higher in the ScH group than in the control group. FT4 and FT3 levels, although in the reference range, were significantly lower in the ScH group at baseline than in the control group. Interestingly, after the thyroid substitution therapy, FT4 and FT3 levels significantly increased in the ScH group (Table 1).

**Table 1:** Demographic, clinical, and hormonal parameters of study population

	Control group (n = 30)	Baseline ScH (n = 54)	ScH after 5 months (n = 54)	Statistical significance
Age (years)	39.3 ± 11.7	43.1±12.4		NS
Sex (m:f)	3 : 27 (10%)	2.52 (3.7%)		NS
BMI (kg/m^2^)	24.3 ± 3.0	26.7 ± 4.2	25.7 ± 4.2	NS
BSA (m^2^)	1.78 ± 0.15	1.79 ± 0.17	1.78 ± 0.16	NS
HR (bpm)	76.4 ± 9.1	73.4 ± 9.8	77.1 ± 9.9	p < 0.05^b^
Systolic BP (mmHg)	107.3 ± 28.4	123.3 ± 16.4	122.1 ± 14.2	p < 0.05^a^,^c^
Diastolic BP (mmHg)	77 ± 13.4	80.0 ± 9.3	79.2 ± 8.4	NS
TSH mU/L	1.7 ± 1.05	8.1 ± 1.3	2.8 ± 2.6	p<0.001^a^,^b^
FT4 pmol/L	15.4 ± 2.2	12.3 ± 2.0	15.2 ± 2.6	p<0.01^a^,^b^
FT3 pmol/L	5.2 ± 2.1	4.5 ± 1.1	6.4 ± 3.3	p < 0.05^a^,^b^

Displayed results are average ± std deviation and percentages. Comparisons between the groups were performed by Student's *t*-test (independent and dependent) for continuous variables and χ2 test for categorical variables.

a Statistical significance for controls *vs.* baseline ScH;

b statistical significance for baseline ScH *vs.* ScH after 5 months;

c statistical significance for controls *vs.* ScH after 5 months.

BMI: body mass index; BSA: body surface area; HR: heart rate; BP: blood pressure; NS: no significance.

The LV diameters and volumes were similar in both groups, ScH at baseline and control groups. Overall, only EF (although in referent range in all analysed groups) statistically significantly increased in the ScH group after the levothyroxine therapy (Table 2).

**Table 2:** Echocardiographic parameters of left ventricular function in the study population

	Control group (n = 30)	Baseline ScH (n = 54)	ScH after 5 months (n = 54)	Statistical significance
LA (mm)	31.7 ± 3.1	31.3 ± 3.9	31.7 ± 3.9	NS
LAVI (ml/m^2^)	24.4 ± 4.31	21.9 ± 5.77	20.7 ± 5.43	NS
LVEDD (mm)	46.0 ± 4.8	46.4 ± 4.3	45.6 ± 4.1	NS
LVED vol (mL^3^)	79.1 ± 11.9	81.7 ± 18.4	79.0 ± 18.6	NS
LVES vol (mL^3^)	31.3 ± 7.0	31.6 ± 7.8	30.6 ± 6.5	NS
EF (%)	62.8 ± 2.3	61.6 ± 4.4	62.9 ± 3.9	p < 0.05^b^
IVS (mm)	10.4 ± 1.1	10.8 ± 0.9	10.8 ± 1.0	NS
PW (mm)	8.7 ± 1.2	8.7 ± 1.2	8.7 ± 1.1	NS
E/A (m/sec)	1.26 ± 0.36	1.03 ± 0.29	1.09 ± 0.34	p<0.01^a^,^c^
DT(msec)	156.8 ± 29.7	167.9 ± 38.6	158.5 ± 32.2	NS
E/e’ sep.	6.04 ± 1.64	7.62 ± 2.29	6.60 ± 2.06	p<0.01^a^,^b^
E/e’ lat.	6.08 ± 1.24	6.35 ± 1.62	6.03 ± 1.74	NS
E/e’ average	6.06 ± 1.24	6.98 ± 1.9	6.74 ± 1.7	NS
IVCT (msec)	60.04 ± 10.9	64.14 ± 13.4	60.29 ± 12.7	NS
IVRT (msec)	66.39 ± 8.3	67.27 ± 13.7	65.0 ± 12.8	NS
MPI	0.43 ± 0.07	0.47 ± 0.08	0.43 ± 0.07	p<0.05^a^;p<0.01^b^
s/d	1.26 ± 0.11	1.26 ± 0.16	1.25 ± 0.22	NS
GLS (%)	−20.9 ± 1.7	−19.55 ± 2.3	−20.07 ± 2.7	p < 0.001^a^; p < 0.05^b^
S/TDI (m/sec)	0.092 ± 0.011	0.077 ± 0.013	0.078 ± 0.01	p<0.01^a^,^c^
Ar-A	−18.87 ± 10.78	−25.2 ± 16.1	−23.4 ± 9.1	NS

Displayed results are average ± std deviation and percentages. Comparisons between the groups were performed by Student's *t*-test (independent and dependent) for continuous variables and χ^2^ test for categorical variables.

a Statistical significance for controls *vs.* baseline ScH;

b Statistical significance for baseline ScH *vs.* ScH after 5 months;

c statistical significance for controls *vs.* ScH after 5 months.

LA: left atrial systolic diameter; LAVI: left atrial volume index; LVEDD: end-diastolic diameters; PW: the left ventricular posterior wall; IVS: interventricular septum thickness; LVESV: left ventricular end-systolic volume; LVEDV: left ventricular end-diastolic volume; EF: ejection fraction; E/A: ratio between transmitral early and late diastolic peak flow velocities; A dur: duration of the atrial contraction; DT: time between E velocity deceleration time to the baseline; S wave obtained by TDI, maximal systolic flow velocity; IVCT: isovolumetric contraction time; IVRT: isovolumetric relaxation time; MPI: myocardial performance index; s: systolic velocity of the pulmonary veins; d: diastolic velocity of the pulmonary veins; their ratio (s/d); Ar: retrograde pulmonary venous flow during the atrial contraction-atrial reversal; A-Ar: difference in time of duration of the A wave from transmitral flow by PW and duration of the Ar wave from pulmonary vein flow; GLS: global longitudinal strain.

The results from echocardiography (systolic and diastolic parameters) in all analysed groups were in normal range, but some of them were statistically different compared to ScH and the control group at baseline, as well as to the ScH groups before and after treatment. The transmitral E/A ratio was lower and E/e’ sep. ratio was higher in the ScH group at baseline than in the control group. The E/e’ sep. ratio significantly decreased in the ScH group after levothyroxine therapy. The MPI was higher in the ScH group at baseline than in the control group and significantly decreased after levothyroxine therapy. The GLS was significantly reduced in the ScH group at baseline in comparison to the control group and significantly improved after levothyroxine therapy. The S wave estimated from TDI was significantly lower in the ScH group at baseline than in the control group, without changes after the levothyroxine therapy (Table 2). Analysing how many patients had diastolic dysfunction, according to new guidelines for the evaluation of diastolic function by echocardiography, showed that there were only three patients with ScH (22). In all three patients diastolic dysfunction was reversible after thyroid replacement therapy.

Considering the entire study population, the TSH levels (before and after levothyroxine therapy) significantly negatively correlated with EF (r = −0.15, p < 0.05), E/A ratio (r = −0.14, p < 0.05), GLS (r = −0.26, p < 0.001), and S/TDI (r = −0.22, p < 0.01) and positively correlated with E/e’ sep. (r = 0.14, p < 0.05).

## DISCUSSION

The results of the study presented subtle changes in the LV systolic and diastolic function which were reversible after thyroid replacement therapy. Although the values of the echocardiography parameters were in normal range in all analysed groups (control, ScH at baseline, and SCH after treatment), there were statistically significant differences between some parameters compared to the control and ScH group at baseline, as well as to the ScH group before and after treatment.

Our results show that the ScH group at baseline had statistically significantly higher values for systolic blood pressure. This result is similar to the results in one meta-analysis published in 2014. The study includes the analysis of 20 studies and found a slight increase in systolic blood pressure in patients with ScH versus the control group (23). Systolic blood pressure did not decrease significantly after the levothyroxine treatment. The average value for systolic blood pressure in the ScH group at baseline was normal (123.3 ± 16.4 mmHg), so the thyroid replacement therapy probably had no clinical significance in reducing its value. Thus, we can conclude that ScH had no significant impact on the arterial blood pressure, in contrast to what is already known about clinical hypothyroidism. Therefore, we do not expect the thyroid replacement therapy to significantly decrease the already normal values for systolic blood pressure.

Heart rate statistically significantly increased after thyroid replacement therapy. These changes are consistent with the known mechanism of action of thyroid hormones. From a clinical point of view, the increase in heart rate after treatment was still in the normal range (77.1 ± 9.9 minute), but there was statistical significance. None of the patients after treatment had tachycardia or arrhythmia.

At baseline, echocardiographic parameters of LV function were not significantly different between the control and the ScH group, which were similar with results from other studies (15,24-28). Five months of levothyroxine therapy statistically significantly increased the EF. Only a small (1.3%) increase in EF was sufficient to make EF equal to the value (62.9 ± 3.9%) as in the control group. In the study of Ilic and cols. (29), one-year therapy with levothyroxine showed results similar to ours. It is important to mention that all studies (including ours) that analyse the EF in patients with ScH have normal values for EF.

The parameter S derived from TDI, which is a parameter to assess systolic function of LV, was statistically significantly lower in the ScH group compared to the control group. This proves the abnormal longitudinal systolic function of LV in ScH, which is first affected in LV systolic dysfunction (30,31). The parameter S/TDI did not recover after treatment, but there was statistically significant correlation between TSH and S/TDI. More aggressive treatment for lowering TSH value may result in statistically significant changes in S/TDI. Completely identical results were shown in the study of Ilic and cols. (29). The average age in the study of Ilic and cols. (29) is similar to ours, but they analysed only women aged less than 45 years. Probably studies with a large number of patients involving only women in reproductive age are needed to confirm the impact of ScH on S wave derived from TDI.

Results for MPI showed deterioration in global systolic and diastolic function of the LV in ScH, and its reversibility after thyroid replacement therapy. Our results were consistent with those from other clinical studies (24,25,29). The average values of TSH in those studies are similar to this study. There was no statistically significant correlation between TSH and MPI. The lack of correlation between TSH and MPI may be due to small changes in the value of MPI that occur at higher values of TSH.

Global longitudinal strain is considered more sensitive than EF in assessing LV global systolic function, especially when the EF is normal, in favour of subclinical LV dysfunction. Values of GLS in the control group and ScH group at baseline were within the normal range, but still with statistically significantly different values in the ScH group. GLS significantly improved after the levothyroxine therapy. TSH significantly negatively correlated with GLS. Two studies examined GLS in ScH, and both of them showed results similar to ours (29,32).

The results from Doppler transmitral flow and velocities estimated by TDI showed changes in some parameters which are defined as parameters for diastolic function assessment. Although the values of some parameters were in the normal range, there is a statistically significant difference between the values of the control and ScH groups. This is shown by statistically significantly lower E/A ratio, and higher E/e’ sep. ratio in the ScH group compared to the control group.

After 5 months of euthyroid state, the E/A ratio did not increase significantly. In a study by Yazici and cols. (25) the E/A ratio was statistically significantly lower in patients with ScH compared to the control group. In the same study, the ratio increased significantly after 6 months of euthyroid state and continued to increase after 12 months of euthyroid state. A study by Erkan and cols. (33) showed results similar to ours. Therefore, a longer euthyroid state or more aggressive treatment for lowering TSH may be required to achieve a statistically significant increase in the E/A ratio, after treatment. At the end of the study the average TSH value was 2.8 ± 2.6 mU/L. In the assessment of diastolic function the E/e’ sep. ratio is a more sensitive parameter than the E/e’ lat. ratio. A statistically significant difference in the E/e’ sep. ratio, but not in the E/e’ lat. Ratio, was found in other studies (24,28). Three patients in the ScH group had diastolic dysfunction, which was reversible after thyroid replacement therapy. Diastolic dysfunction is mainly attributed to ScH because other potential causes of diastolic dysfunction (hypertension, diabetes mellitus) were excluded in this study. Parameters which measured diastolic dysfunction can be applied to all patients with ScH and also to follow them during treatment.

Statistically significant correlations between TSH and analysed parameters were weak (r < 0.5). The weak correlation coefficient may be due to higher TSH values at the end of the study. The r values were lower for correlations with diastolic parameters. Probably systolic parameters are more sensitive on L-T4 treatment, and diastolic parameters need a longer period of treatment.

Summarizing the results, ScH did not cause cardiac failure by itself, but thyroid substitution therapy improves some parameters of LV function. We analysed only patients with mild ScH, which means rapid detection of thyroid dysfunction. If ScH is detected later, according to the correlations, we expected worsening of LV parameters.

There are some limitations in this study. The design is not that of a blind or double-blind study which includes L-thyroxine and placebo. We followed the parameters of systolic and diastolic function, but we did not directly observe cardiovascular morbidity and mortality, which requires very long-term monitoring of patients.

The advantages of the study compared to previous similar studies are the inclusion of young people with no risk factors for cardiovascular disease which can influence systolic and diastolic parameters, and the inclusion of patients with mild ScH. Thus, this study confirmed the benefit of levothyroxine treatment in patients with ScH with lower values of TSH. Today, when there is a lack of recommendations for treatment of mild ScH, this study moves the limits for initiation with treatment from the lower values of TSH.

In conclusion, patients with ScH versus healthy individuals had subtle changes in certain parameters that indicate involvement of systolic and diastolic function of the LV in ScH. Although the values of the parameters were in normal range, they were significantly different compared to ScH and the control group at baseline, as well as to the ScH groups before and after treatment. The results of our study suggest that the patients with ScH deserve to be followed up during treatment.
